# Interleukin-7 Influences FOXP3^+^CD4^+^ Regulatory T Cells Peripheral Homeostasis

**DOI:** 10.1371/journal.pone.0036596

**Published:** 2012-05-07

**Authors:** Federico Simonetta, Nicolas Gestermann, Kim Zita Martinet, Michele Boniotto, Pierre Tissières, Benedict Seddon, Christine Bourgeois

**Affiliations:** 1 INSERM, Le Kremlin-Bicêtre, France; 2 Université of Paris, Le Kremlin-Bicêtre, France; 3 Division of Immune Cell Biology, National Institute for Medical Research, The Ridgeway, London, United Kingdom; Otto-von-Guericke University Magdeburg, Germany

## Abstract

Mechanisms governing peripheral CD4+ FOXP3+ regulatory T cells (Treg) survival and homeostasis are multiple suggesting tight and complex regulation of regulatory T cells homeostasis. Some specific factors, such as TGF-β, interleukin-2 (IL-2) and B7 costimulatory molecules have been identified as essentials for maintenance of the peripheral Treg compartment. Conversely, Treg dependency upon classical T cell homeostatic factors such as IL-7 is still unclear. In this work, we formally investigated the role of IL-7 in Treg homeostasis *in vivo* in murine models. We demonstrated that IL-7 availability regulated the size of peripheral Treg cell pool and thus paralleled the impact of IL-7 on conventional T cell pool. Moreover, we showed that IL-7 administration increased Treg cell numbers by inducing thymic-independent Treg peripheral expansion. Importantly the impact of IL-7 on Treg expansion was detected whether conventional T cells were present or absent as IL-7 directly participates to the peripheral expansion of Treg after adoptive transfer into lymphopenic hosts. Our results definitively identify IL-7 as a central factor contributing to Treg peripheral homeostasis, thus reassembling Treg to other T cell subsets in respect of their need for IL-7 for their peripheral maintenance.

## Introduction

CD4^+^ CD25^+^ FOXP3^+^ regulatory T cells are a population of CD4 T cells crucial for the regulation of immune responses and in preventing autoimmunity and chronic inflammation [1]. Mice and humans genetically deficient in Treg cells as a result of mutations in Foxp3, a critical transcription factor for Treg development and function, typically present severe lympho-proliferation and immune pathology [2]–[5]. Treg exert their major role in the maintenance of immune-tolerance through several mechanisms including secretion of inhibitory molecules, suppression of antigen-presenting cells function, cytolysis and effector cells metabolic disruption [6]. Despite the importance of the Treg compartment for the maintenance of immune tolerance and the intensive investigation focusing on Treg cell biology during the last few years, uncertainties remain about the factors controlling peripheral Treg survival and homeostasis. While Treg homeostasis appears to rely on some subset specific factors such as TGF-β [7]–[9], IL-2 [10]–[13] and B7 costimulatory molecules [14], [15], their dependency upon factors classically involved in conventional T cells homeostasis such as IL-7 signaling still remains controversial. IL-7 plays a critical role in T cell development and peripheral homeostasis [16]. All major CD4 T cell subsets, including naïve, memory and Th17 CD4 T cells, strictly rely on IL-7 for their peripheral homeostasis [17]–[20]. The only important exception seems to be represented by CD4^+^ FOXP3^+^ Treg which have been reported to express low levels of the IL-7 receptor alpha chain (CD127) [21]–[23]. Accordingly, peripheral Treg biology is believed to be essentially independent of IL-7/IL-7Rα signaling [17], [24]–[28] although Bayer *et al* suggested that IL-7 could contribute to Treg homeostasis when IL-2 signaling is disrupted [24]. However, we and others have previously shown that *in vitro* IL-7 treatment induces significant STAT5 phosphorylation in Tregs [26], [29]–[31] and that IL-7 is able to increase *in vitro* Treg survival [30], [32], [33]. These *in vitro* data revealing a potential role of IL-7 on Treg homeostasis need to be substantiated by *in vivo* analysis. We first considered Treg homeostasis in mice exhibiting altered IL-7 signaling pathway using IL-7Rα−/−, IL-7−/− or IL-7 Tg mice. Regarding the increasing development of IL-7 based therapies we also investigated how *in vivo* Treg homeostasis was affected following IL-7 injection. We demonstrated that IL-7 availability regulated the size of the peripheral Treg cell pool and that Treg and Tconv were equally affected following IL-7 injection. Moreover, we showed that IL-7 administration increased Treg cell numbers by inducing a thymic-independent peripheral expansion. Importantly the impact of IL-7 on Treg expansion was detected whether conventional T cells were present or absent as IL-7 directly participates to the peripheral expansion of Treg after adoptive transfer into lymphopenic hosts. Collectively, our data identify IL-7 as a central factor contributing to Treg peripheral homeostasis. Such a conclusion has major implications for the development of IL-7 based strategies to ameliorate immune-reconstitution in lymphopenic settings while preventing immune pathology.

## Results

### IL-7 availability affects the size of the conventional and regulatory T cell pools to a similar extent

**Figure 1:**
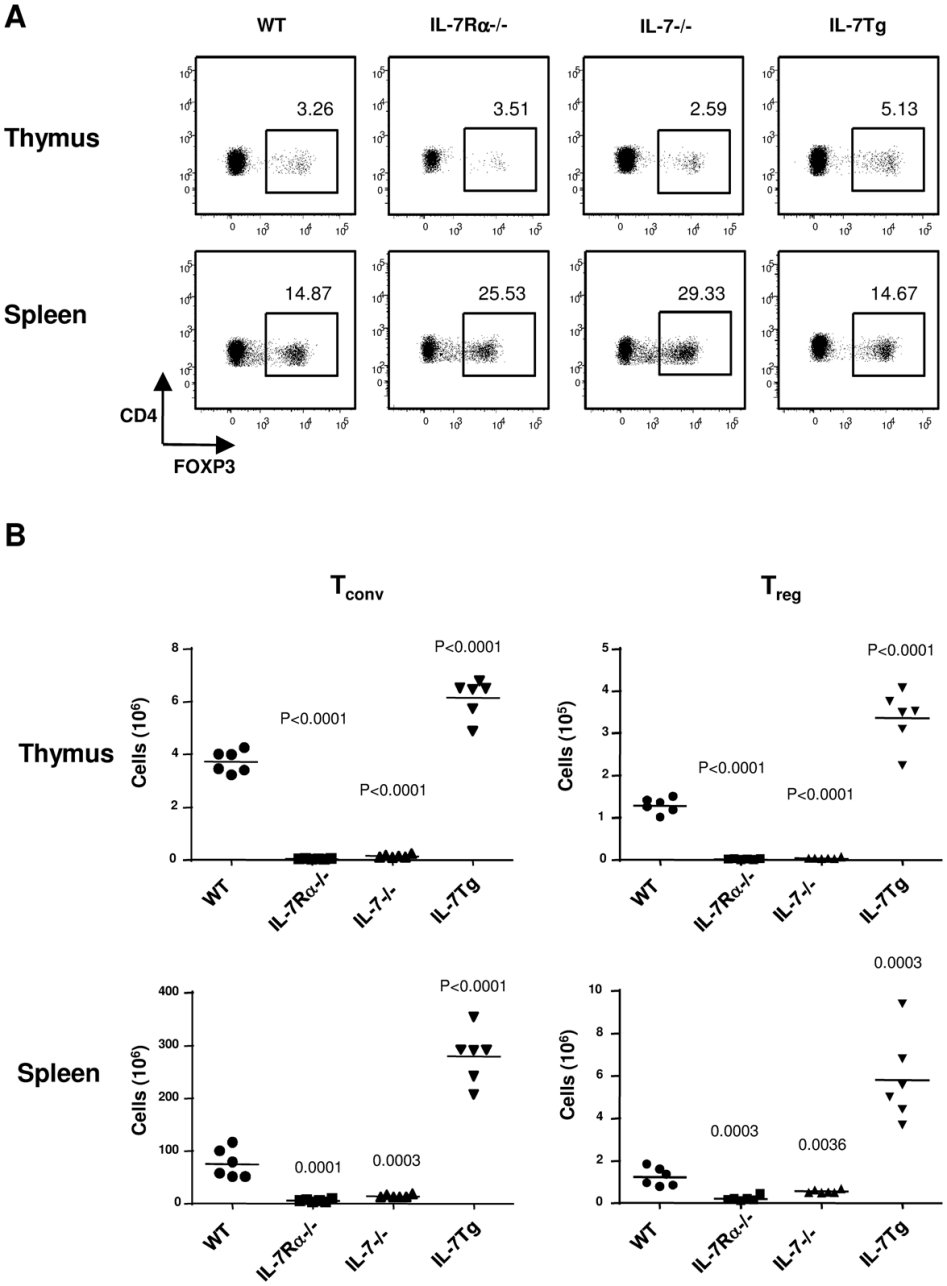
IL-7 signaling is involved in both thymic development and peripheral maintenance of Foxp3+ Treg. (A) Representative dot plots of FOXP3 expressing cells gated on CD4^+^ CD8^−^ TCRβ^+^ thymocytes and splenic CD4^+^ TCRβ^+^ cells from normal B6, IL-*7*Rα−/−, IL-7−/− and IL-7 Tg animals. Numbers in each dot plot indicate the relative percentages of FOXP3 cells among CD4^+^ T cells for representative animal selected among 4 to 6 mice per group. (B) Absolute numbers of CD4^+^ FOXP3^+^ T cells from normal B6, IL-*7*Rα−/−, IL-7−/− and IL-7 Tg animals. Each point represents a single mouse and mean values are indicated for each groups. Experiments were repeated independently twice with four to six mice per group; the data shown are representative of one experiment. The degree of statistical significance (p values), relative to normal B6 numbers, is indicated.

To provide an exhaustive analysis of the influence of IL-7 on Treg homeostasis *in vivo*, we first examined mice in which IL-7 signaling was genetically disrupted (IL-7Rα−/− or IL-7−/−) or increased (IL-7 Tg). Because IL-7 directly impacts thymic cellularity, both thymic and peripheral Treg distribution and cellularity were determined. In agreement with previous reports [26], [27], IL-7Rα−/− mice display similar percentages of thymic FOXP3^+^ cells among single positive CD4 cells when compared to normal C57Bl/6 mice (3.43%±0.76% in IL-7Rα−/− versus 3.33%±0.24% in WT) while IL7−/− mice present slight though statistically significant reduction in proportion of FOXP3^+^ cells among CD4 T cells (2.62±0.23%, p 0.003) (Fig. 1A). Conversely, significantly higher percentages of Treg were observed in thymi from IL-7 Tg mice (5.16±0.48%, p<0.0001) (Fig. 1A). Study of absolute thymic T cell numbers revealed that the expected reduction in FOXP3^−^ CD4^+^ single positive thymocytes in IL-7Rα−/− or IL-7−/− mice was associated to a proportional reduction in the FOXP3^+^ CD4^+^ single positive compartment (Fig. 1B). Accordingly IL-7 over-expressing mice presented higher numbers of both FOXP3^−^ and FOXP3^+^ CD4^+^ single positive thymocytes. Collectively these data suggest that IL-7 signaling similarly affects conventional T cells (Tconv) and Treg cell thymic development. We then focused on splenocytes in order to evaluate IL-7 contribution in controlling Treg cell peripheral cell numbers. As already described, higher percentages of FOXP3^+^ cells were found among splenic CD4 T cells from IL-7Rα−/− and IL-7−/− mice when compared to control C57Bl/6 mice (Fig. 1A) (27.18%±4.23% in IL-7Rα−/− and 28.26%±0.99% in IL-7−/− versus 14.09%±1.09% in WT; p<0.0001). Such a difference is presumably linked to impaired peripheral survival of conventional naïve CD4 T cells in the absence of IL-7 signaling and suggests a lower impact of IL-7 on Treg. Interestingly, IL-7 Tg mice presented preserved proportion of splenic Treg cells among CD4 T cells (17%±3.62%) (Fig. 1A). Analyzing T cells numbers, we observed as expected that conventional CD4^+^ FOXP3^−^ T cell numbers were significantly reduced in IL-7Rα−/− and IL-7−/− mice (13.8-fold and 5.4-fold reduction respectively) while they were present in higher numbers in IL-7 Tg mice (WT 7.5×10^6^±2.8×10^6^ vs IL-7 Tg 27.9×10^6^±5.0×10^6^, p<0.0001) (Fig. 1B). Accordingly, IL-7Rα−/− and IL-7−/− mice presented significantly reduced but yet substantial numbers of peripheral Treg cells compared with their wild type counterparts (WT 1.2×10^6^±0.4×10^6^ vs IL-7Rα−/− 0.2×10^6^±0.1×10^6^ vs IL-7−/− 0.5×10^6^±0.1×10^6^), while IL-7 Tg mice presented a 4.7-fold increase in Treg cell number (Fig. 1B). Interestingly the extent of Treg number variation in spleen from KO or Tg mice reflected the variation we observed in the conventional FOXP3^−^ CD4 T cell compartment. Due to the crucial role of IL-7 on thymic development, these results do not allow drawing any conclusion on the direct impact of IL-7 on peripheral T cell homeostasis. Nevertheless, these results suggest that IL-7 similarly affects conventional and regulatory T cell numbers by acting on thymic development and/or peripheral homeostasis.

### IL-7/αIL-7 complexes treatment induces both conventional and regulatory T cells expansion

**Figure 2:**
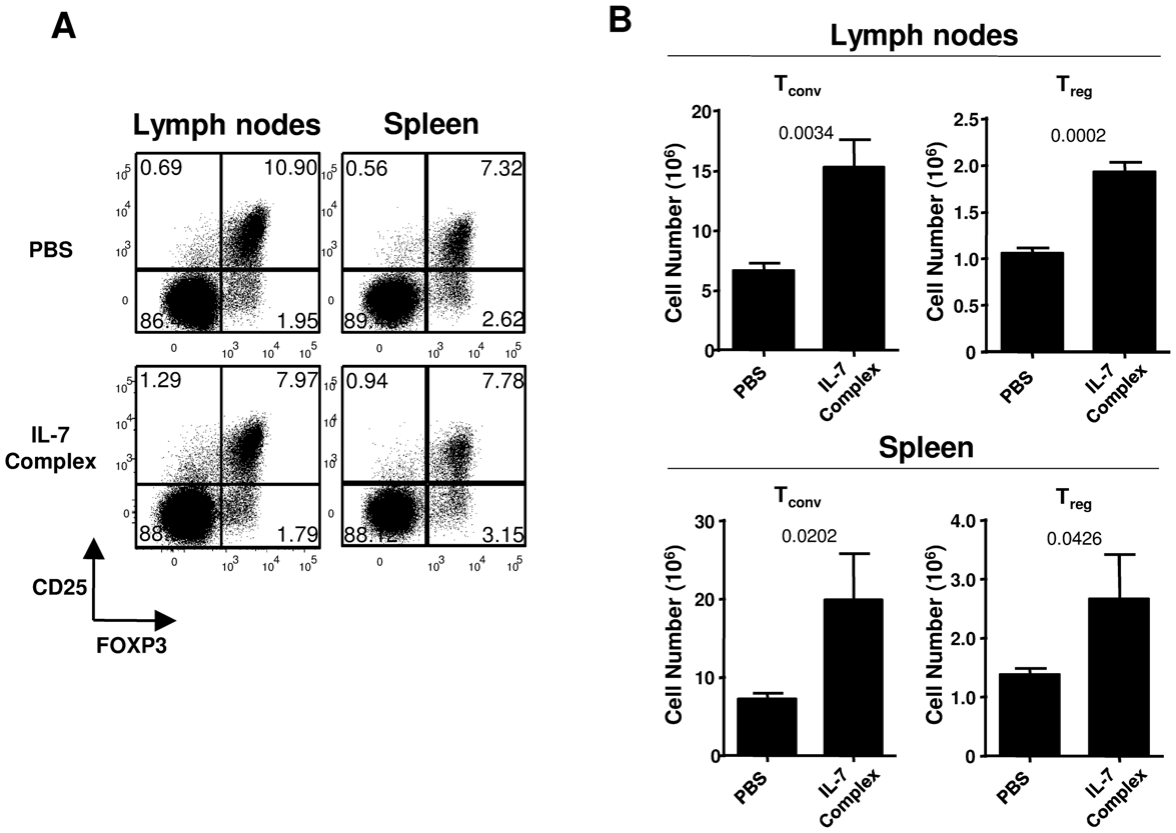
IL-7 complexes treatment induced both conventional and regulatory T cell expansion. C57Bl/6 mice were i.p. injected with PBS or IL-7/anti-IL-7 complexes (1 µg rmIL-7 plus 5 µg M25) three times at 2 day intervals. LN and SP cells were analyzed 7 days after the first injection. (A) Representative dot plots of CD25 vs FOXP3 expression on CD4^+^TCRβ^+^ cells recovered from PBS or IL-7 complexes treated mice. (B) Total number of CD4^+^TCRβFOXP3^−^ conventional T cells (left panels) and CD4^+^ TCRβ FOXP3^+^ regulatory T cells (right panels) from lymph nodes and spleen.

**Figure 3:**
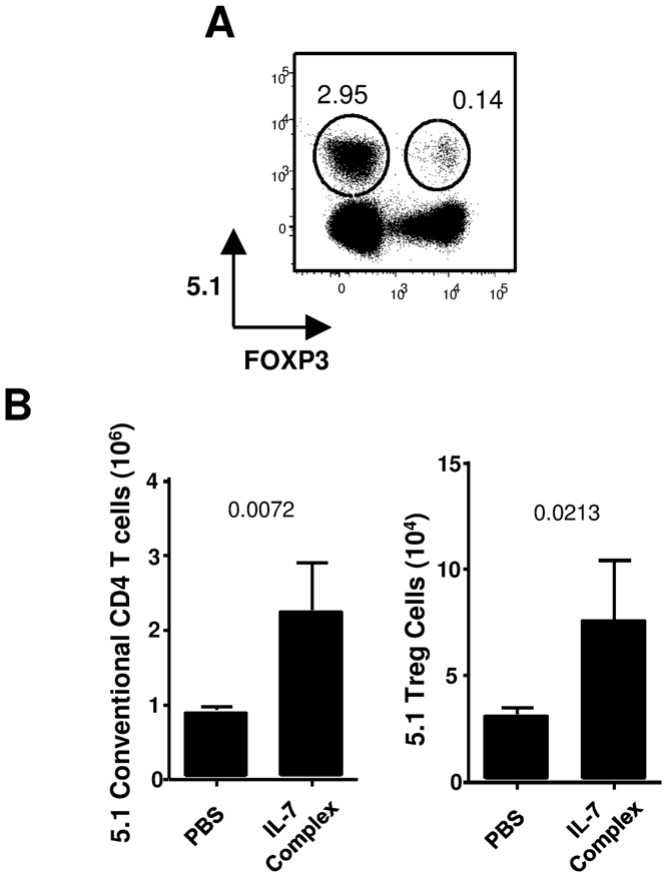
IL-7 induced expansion of both conventional and regulatory T cells is thymic independent. CFSE labeled total CD4 T cells from CD45.1 mice were transferred into CD45.2 C57Bl/6 hosts. Mice were then treated with IL-7/anti-IL-7 complexes as in Fig. 2. (A) Representative dot plots of CD45.1 vs FOXP3 expression on CD4^+^ TCRβ cells recovered from lymph nodes and spleens of PBS or IL-7 complexes treated mice. (B) Total numbers of adoptively transferred CD45.1 FOXP3^−^ conventional T cells (left panels) and CD45.1 FOXP3^+^ regulatory T cells (right panels) from lymph nodes and spleen.

To better assess the peripheral role of IL-7, we determined the effects of exogenous IL-7 treatment on Treg cells. C57Bl/6 mice were injected with IL-7/αIL-7 complexes or PBS at d0, d2 and d4. Such a treatment protocol has been reported to induce strong peripheral expansion of conventional CD4 and CD8 T cells in non-lymphopenic hosts [25]. Flow cytometry analysis at day 7 from the first injection revealed a slight but significant reduction in the percentage of FOXP3^+^ CD4 T cells in the lymph-nodes (NT 11.1%±1.0% vs IL-7 9.2%±1.1%, p = 0.044) that was not detected in the spleen (NT 10.8%±1.4% vs IL-7 11.4%±0.6%, p = 0.586) (Fig. 2A). When considering absolute numbers, IL-7 treatment increased both CD4^+^ FOXP3^−^ and FOXP3^+^ cell numbers (approximately 2 fold increase in both LN and SP) (Fig. 2B). Similarly to the analysis in mice bearing disrupted or increased IL-7 signaling (Fig. 1), conventional and regulatory T cells thus appeared similarly affected by IL-7 injection treatment. Theoretically, increase in peripheral T cell numbers results from effects on peripheral proliferation, cell survival and/or thymic production. Total and Treg thymic cellularity were not significantly different in IL-7/αIL-7 treated animals compared to PBS injected animals (data not shown). To exclude any potential contribution of thymopoiesis on observed IL-7 effects, we adoptively transferred CFSE stained CD4 T cells from CD45.1 mice into CD45.2 C57Bl/6 mice prior to IL-7 complexes treatment. Use of CD45.1 and FOXP3 staining allowed us to distinguish progeny of adoptively transferred cells from host cells (Fig. 3A). At day 7, a two-fold expansion was observed in FOXP3^+^ CD4^+^ transferred CD45.1 Treg cells isolated from IL-7 complexes treated mice when compared to PBS injected mice (Fig. 3B). Collectively, these data demonstrate that IL-7 impacts peripheral Treg homeostasis.

### IL-7/αIL-7 complexes treatment affects the Treg proliferative fraction

We next questioned whether IL-7 treatment affected Treg proliferation as described for conventional T cells.

**Figure 4:**
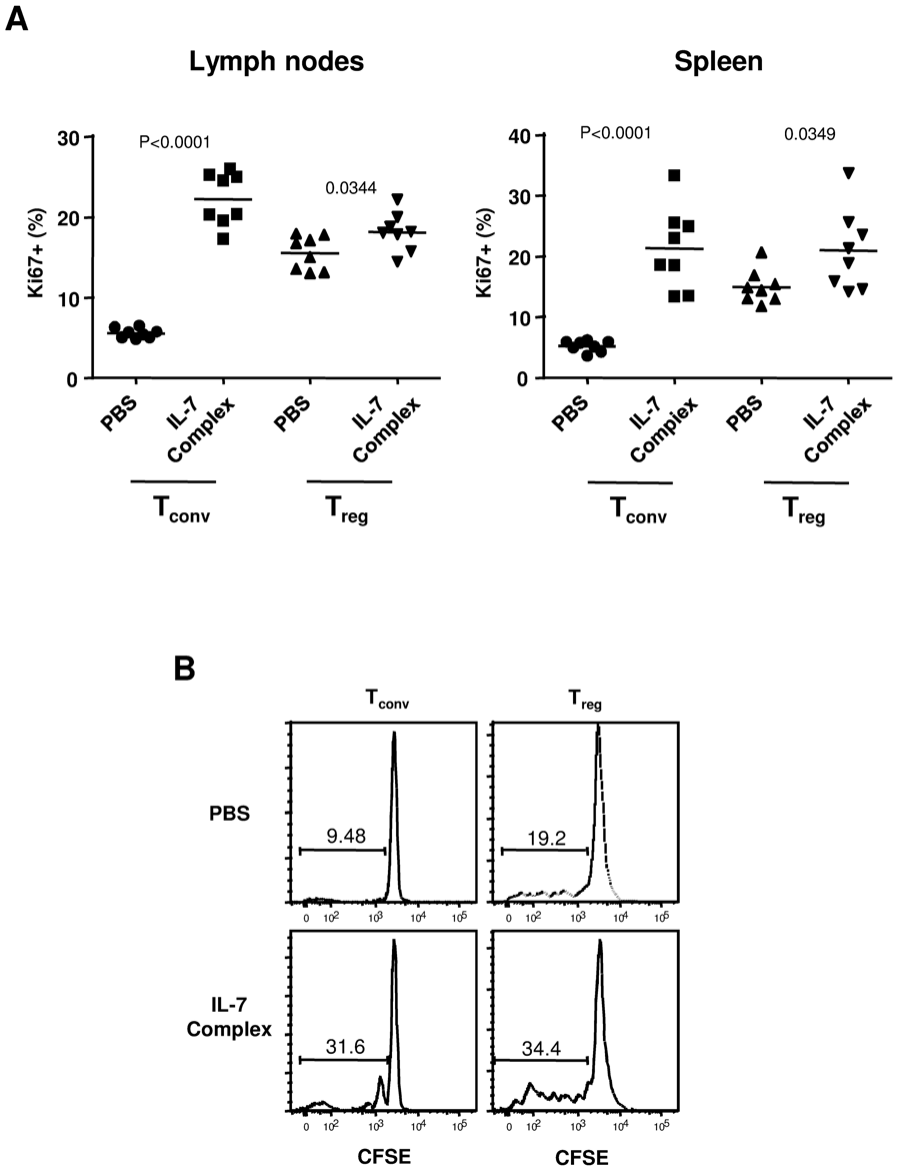
IL-7 mildly impacts CD4+CD25+ Treg cells proliferative capacity. Unmanipulated C57Bl/6 mice (A) or C57Bl/6 mice transferred with CFSE labeled total CD45.1 CD4 T cells (B) were i.p. injected with PBS or IL-7/anti-IL-7 complexes (1 µg rmIL-7 plus 5 µg M25) three times at 2 day intervals. (A) Percentage of cells expressing the proliferation marker Ki67 in CD4^+^ TCRβ^+^FOXP3^−^ conventional T cells and CD4^+^ TCRβ FOXP3^+^ regulatory T cells from LN (left panel) and spleen (right panel). Percentage of Ki67 positive cells were determined based on isotype control. Results were pooled from 2 separate experiments, using three to four mice per group. (B) Representative CFSE profiles of CD45.1 Tconv and Treg isolated at day 7 from PBS or IL-7 complexes treated mice. Experiments were repeated independently three times with two to four mice per group. Data shown are representative of one experiment.

We examined Ki67 staining, which identifies proliferating cells, in C57Bl/6 mice injected or not with IL-7/αIL-7 complexes. The fraction of cycling conventional CD4 T cells was 4 fold increased in lymph nodes reaching 22.3%±3.3% of conventional T cells, whereas the Treg proliferating fraction was slightly but significantly affected (NT 15.5%±2.1% vs IL-7 18.2%±2.4%, p = 0.0344) (Fig. 4A). Similar observation was obtained when analyzing splenic conventional T cells and Treg cells. We next evaluated the contribution of peripheral cell proliferation in the observed expansion of CD45.1 T cells transferred into C57Bl/6 mice, as described in Fig. 3. We analyzed the CFSE dilution profile of adoptively transferred cells isolated from LN at day 7. IL-7 complexes treatment induced an increase in the CFSElow proliferating fraction in both conventional (NT 5.8%±0.6% vs IL-7 18.8%±8.2%, p = 0.0191) and Treg cell (NT 17.7%±3.7% vs IL-7 26.5%±4.6%, p = 0.0252) compartments (Fig. 4B). Similar observation was obtained when splenic conventional and Treg cells were analyzed. In accordance to data obtained with the Ki67 staining, increase in proliferation upon IL-7 treatment was more important in Tconv than in Treg cell compartments. Collectively, these results indicate that IL-7 therapy affects both conventional and regulatory T cell numbers and acts at different extent on their proliferation.

### IL-7 directly induces Treg peripheral expansion in lymphopenic hosts

**Figure 5:**
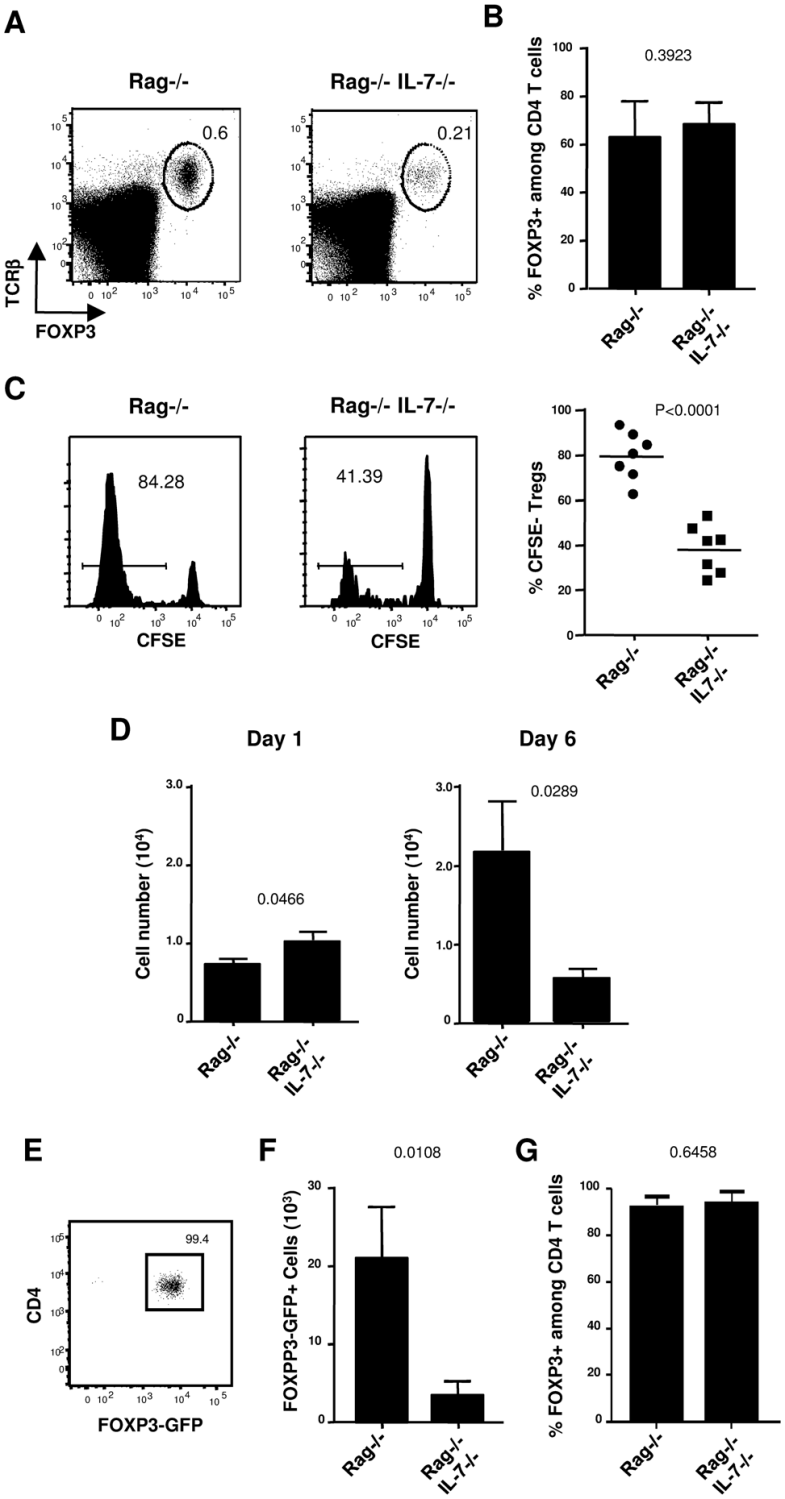
IL-7 promoted expansion of FOXP3+ Treg cells in lymphopenic hosts. (**A, B, C**) 1.10^6^ CFSE labeled, purified CD4^+^CD25^+^ T cells were transferred into Rag−/− and Rag−/− IL-7−/− mice. (A) Representative dot plots of FOXP3 vs TCRβ expression on splenic lymphocytes at day 6 after transfer. (B) Percentages of FOXP3^+^ cells among CD4^+^ TCRβ^+^ cells at day 6 after transfer. (C) Representative profiles of CFSE dilution of CD4^+^ TCRβ^+^ FOXP3^+^ cells at day 6 after transfer, and graph of CFSElow Tregs percentage recovered from three experiments using two to three mice per group. (D) Total splenic numbers of CD4^+^TCRβ^+^FOXP3^+^ cells recovered per mouse at day 1 and 6. (E,F,G) 5 10^5^ CD4^+^ GFP^+^ cells isolated from Foxp3GFP mice were transferred into Rag−/− and Rag−/−IL-7−/− mice. (E) Representative dot plots of FOXP3GFP vs CD4 expression in FACS sorted Treg cells before transfer. (F) Total splenic numbers of CD4^+^TCRβ^+^FOXP3GFP^+^ cells recovered per mouse at day 8. (G) Percentages of FOXP3GFP^+^ cells among CD4^+^TCRβ^+^ cells at day 8 after transfer. Experiments were repeated twice with three mice per group. Data shown are representative of one experiment.

Treg survival is highly dependent on IL-2 [12], [13]. Increase in Treg cellularity upon IL-7 treatment may reflect increased IL-2 production by the proliferating conventional/effector cells fraction. To formally address the direct impact of IL-7 on Treg biology, we used adoptive transfer into lymphopenic hosts, in which Treg expansion could be observed in the absence of potential IL-2 producing conventional T cells. We first transferred purified CFSE labeled CD4^+^ CD25^+^ cells into IL-7 competent or deficient empty hosts (Rag−/− or Rag−/− IL-7−/− mice) (Fig. 5). Treg recovered from spleen of both groups were identified by FOXP3 intracellular staining at day 1 and 6 (Fig. 5A). Importantly, similar proportions of FOXP3^−^ non Treg cells were detected in the two groups of recipient mice suggesting similar impact of potential contaminating effector T cells (Fig. 5B). We first analyzed the CFSE dilution profile of injected Tregs in both hosts. Higher proportion of dividing FOXP3^+^ Tregs was evident in IL-7 competent hosts compared to IL-7 deficient hosts (Fig. 5C). However, number of division undergone by Tregs did not significantly differ: most dividing Tregs have undergone more than 7 divisions in both Rag−/− and Rag−/− IL-7−/− recipients. We next analyzed absolute Treg cell recovery from recipients spleen. At day 1, Treg cell numbers recovered from spleen were slightly but statistically different between IL-7 deficient and IL-7 competent hosts. Such higher splenic homing in Rag−/− IL-7−/− recipients presumably reflects the absence of LN structures in the absence of IL-7. Conversely, Treg cells numbers recovered at day 6 were significantly higher in Rag−/− hosts compared to Rag−/− IL-7−/− hosts (Fig. 5B). Notably, day 6 Treg recovery in IL-7 competent hosts was increased compared to day 1, whereas Treg numbers recovered in IL-7 deficient hosts were reduced compared to d1 values. In order to confirm our results while excluding the potential bias induced by co-transfer of contaminating conventional non-Treg FOXP3- cells, we adoptively transferred highly purified FACS sorted CD4^+^ FOXP3GFP^+^ cells (Fig. 5E) from mice in which a green fluorescent protein (GFP) encoding sequence was introduced under the FOXP3 promoter (Fig. 5E). Even in these conditions, significantly higher numbers of CD4^+^ TCRβ^+^ FOXP3^+^ Treg cells were recovered in Rag−/− hosts compared to Rag−/− IL-7−/− hosts (Fig. 5F) pointing to a direct effect of IL-7. In this setting where contamination by non FOXP3 cells can be excluded, we detected a small fraction of FOXP3 non expressing T cells at day 8. Down regulation of FOXP3 expression by Treg has been previously described following transfer into empty hosts [34]–[37]. We questioned whether IL-7 availability may affect differently Treg conversion and indirectly modulate Treg accumulation. We thus determined the percentage of Treg conversion in both hosts. At day 8, 93%±2% of CD4^+^ TCRβ^+^ cells recovered from Rag−/− hosts still expressed FOXP3GFP, suggesting limited conversion to effector FOXP3^−^ non-Treg cells (Fig. 5G). Importantly, we did not detect any significant differences in the percentage of converted Tregs in the presence of absence of IL-7 suggesting IL-7 did not crucially affected Treg conversion (7.17%±2.19% in Rag−/− vs 5.53%±2.45% in Rag−/−IL-7−/−; p 0.6458). Collectively, these data demonstrate that Treg expansion and accumulation in lymphopenic settings is IL-7 dependent.

## Discussion

All major CD4 T cell subsets, including naïve, memory and Th17 CD4 T cells, strictly rely on IL-7 for their peripheral homeostasis. The only important exception seems to be represented by CD4^+^ FOXP3^+^ Treg cells, which are believed to essentially rely on other factors such as IL-2, TGF-β and costimulatory molecules. Although several γc cytokines, including IL-2, IL-4, IL-7, IL-15, IL-21, have been reported to increase Treg survival *in vitro* [33], only IL-2 has been clearly established as a major factor controlling Treg cell homeostasis *in vivo* [10]–[13], [38], [39]. Several reports investigated the role of IL-7 on thymic Treg development [24], [26], [27] and tried to extend the analysis to the peripheral effects of IL-7. Using adoptive transfer of total CD4 T cells into irradiated Rag−/− or Rag−/− IL-7−/− mice, Mazzuchelli et al, failed to detect any differences in Treg numbers recovered concluding that IL-7 availability does not affect Treg peripheral survival [26]. Unfortunately, total lymph-node cells were transferred, impeding any conclusions on the direct effect of IL-7 on Treg cells in this setting. The present study aimed to further investigate the effects of IL-7 on the homeostasis of CD25^+^ FOXP3^+^ regulatory T cells. Importantly, we demonstrated that IL-7 availability controls the size of both Tconv and Treg pools. Mice in which IL-7 signaling was impaired presented a reduction in Treg cell numbers similar to those observed in conventional T cell numbers. Conversely, IL-7 Tg mice, in which IL-7 production is constantly increased, showed an expansion of the Treg compartment. However, one may question the relevance of studies performed in mice exhibiting disruption of IL-7/IL-7R signaling, which severely impact thymic differentiation and peripheral T cell homeostasis. We next used a more relevant model allowing us determining the impact of IL-7 on physiologically generated Treg. Using IL-7 injection protocol, we confirm the impact of IL-7 on Treg homeostasis. IL-7 treatment in normal mice induced an increase in conventional CD4 T cell numbers that was strictly paralleled by an increase in Treg cell numbers. Importantly demonstrating that IL-7 levels equally affect the size of conventional and Treg subsets perfectly corroborate results obtained in humans during clinical trials investigating the effects of IL-7 therapy on immune-reconstitution in lymphopenic settings. Indeed, three published reports analyzed the proportion of Treg cells in peripheral blood from patients treated with IL-7 [40]–[42] and detected either slight reduction or no difference in the percentage of Treg cells among CD4 T cells after IL-7 treatment. Because total CD4 T cell count increased in IL-7 treated patients, essentially preserved Treg percentages indicate that Treg changes in absolute numbers also paralleled those detected in conventional CD4 T cells in human studies. We next aimed to determine the mechanisms involved in IL-7 mediated effects on Treg pool. Because the impact of IL-7 on conventional T cell proliferation [43] and survival [44] has been extensively described, we aimed to determine whether IL-7 influenced Treg homeostasis by similar mechanisms. Interestingly, IL-7 differently impacted conventional and regulatory T cell compartment: the fraction of proliferating T cells were 3-fold increased in conventional T cells, but mildly increased in Treg fraction in IL-7 treated C57Bl/6 mice. Although significant, such low increases in Treg proliferative capacity is unlikely to account for the highly significant increase in Treg cellularity upon IL-7/αIL-7 complex treatment. These results thus suggest IL-7 treatment is likely to increase Treg cell numbers in the periphery by affecting both their survival and accumulation as previously suggested [30], [32], [33] together with their proliferative capacity. Because the fraction of proliferating conventional T cells was significantly increased upon IL-7 treatment, we speculated that the beneficial impact of IL-7 on Treg homeostasis essentially rely on increased IL-2 availability due to the increased fraction of activated/effector conventional T cells. Indeed, IL-7 is highly heterogeneous in targets and mechanisms [16], favoring both conventional T cell homeostasis and effector differentiation [45], [46]. To determine the impact of IL-7 on Tregs in the absence of conventional T cells, we performed adoptive transfer of purified Treg into Rag−/− or Rag−/− IL-7−/− mice. We showed that Treg cells transferred into Rag-deficient mice lacking IL-7 proliferated and accumulated less than Treg transferred into IL-7 competent Rag-deficient mice. To note, residual proliferation was still present in the absence of IL-7 indicating that other factors participate to the phenomenon. Although residual IL-2 production by DCs or “exFOXP3” converted Treg cannot be formally excluded [37], [47], other γc cytokines may also impact Treg peripheral homeostasis [33]. We thus demonstrated the direct impact of IL-7 on Treg biology in the absence of conventional T cells. However, we cannot exclude that the impact of IL-7 on Treg homeostasis may combine both direct and indirect effects [48].

In summary, our data establish a role for IL-7 in Treg homeostasis, thus reassembling Treg to other T cell subsets in respect of their need for IL-7 for their peripheral maintenance. They indicate that IL-7 availability similarly affects both the conventional and the regulatory T cell pool, maintaining their proportions relatively unchanged. This balanced effect of IL-7 on both the conventional and the regulatory T cell pool can be important to prevent immune-pathology during both spontaneous and therapeutic IL-7 induced immune reconstitution.

## Materials and Methods

### Ethics Statement

Mice were kept under specific pathogen free conditions in accordance with institutional guidelines in compliance with French and European animal welfare regulations (agreement B-94-043-12, delivered by the French veterinary authorities). All animal studies were approved by the ethics Committee of University Paris Sud and protocols conducted were authorized by French veterinary authorities (licence 94-440) in accordance with French and European guidelines.

### Mice

6 to 8 weeks old C57Bl/6 were purchased from Charles River (Janvier). Mice deficient for Rag gene (Rag−/−) or for Rag and IL-7 genes (Rag−/− IL-7−/−) were used as hosts for adoptive transfer experiments. IL-7Rα−/− mice were purchased from the Jackson Laboratory. IL-7−/− and IL-7 transgenic (IL-7 Tg) mice were kindly provided by Dr. Sophie Ezine. Foxp3-GFP mice were purchased from the Jackson Laboratory. All mice were kept under specific pathogen free conditions and all experiments were performed according to institutional guidelines of the European Community.

### Cell purification

Single cell suspensions were prepared from lymph nodes (pooled inguinal, brachial, axillary and mesenteric) and spleens HBSS containing 2% FCS (PAA Laboratories GmbH). For total CD4 T cells transfer, CD4 T cells were isolated using BD magnetic beads (Becton-Dickinson). For CD4+ CD25+ Treg adoptive transfer experiments, CD4+ CD25+ cells were isolated by magnetic beads accordingly to manufacturer instructions (Miltenyi). Cell purity for FOXP3 was >90%. For CD4+ FOXP3+ Treg adoptive transfer experiments, CD4+ FOXP3GFP+ cells were FACS sorted from Foxp3-GFP mice (Jackson Laboratory) on a FACSAria cell sorter (BD Biosciences). Cell purity for FOXP3 was >99%.

### FACS analysis

Extracellular staining was preceded by incubation with purified anti-CD16/32 antibodies (FcgRII/III block, 2.4G2) (eBioscience) to block nonspecific staining. Cells were stained with FITC-, PE-, PECy5-, PECy7-APC- APCAlexa750- or APC-H7-labeled or biotinylated appropriate antibodies including: CD4 (GK1.5); TCRβ (H57-597); CD25 (PC61.5) or appropriate isotype Abs. Streptavidin-FITC, PECy5 or PECy7 were used to develop biotinylated Abs. All Abs were purchased from eBioscience except APC-H7-labeled antibodies (BD Bioscience). Intranuclear FOXP3 staining was performed using eBioscience PE- or APC conjugated FOXP3 staining buffer set (FJK-16s). Intracellular Ki-67 staining was performed using BD Pharmingen Ki-67 (B56) on permeabilized cells. Six-color flow cytometry was performed with a FACSCanto cytometer (BD Biosciences) and data files were analyzed using FlowJo software (Tree star Inc).

### CFSE labelling

When required, cell division of transferred T cells was assessed by CFSE labelling (Sigma) using standard methods. Cells were resuspended in PBS in a concentration of 10^7^/mL and incubated with CFSE at final concentration of 5 µM for 10 min at 37°C, followed by two washes in ice cold HBSS containing 10% FCS.

### Cytokines

Recombinant murine IL-7 (rmIL-7) was purchased from Immunotools and anti-IL-7 mAb M25 was purchased from BioXcell. Before injection, cytokines/antibody complexes were generated as previously described [25]. Briefly, 1 µg of cytokine was co-incubated with 5 µg of the specific monoclonal antibodies for 30 min at +37°C. Mice were i.p. injected with PBS or IL-7/anti-IL-7 complexes (1 µg rmIL-7 plus 5 µg M25) three times at 2 day intervals.

### Adoptive transfer experiments

For adoptive transfer experiments into wild type mice, total CD4 T cells (7.5×10^6^) from CD45.1 mice were transferred into CD45.2 C57Bl/6 hosts. Mice were then treated with IL-7/anti-IL-7 complexes as described. For adoptive transfer experiments into empty mice, purified CD4+ CD25+ cells (1×10^6^) or CD4+ FOXP3GFP+ cells (5×10^5^) were injected into Rag-deficient mice (Rag−/−) or Rag−/− mice that were also deficient for IL-7 (Rag−/− IL-7−/−). One and/or six to eight days after transfer, spleens were harvested and analyzed.

### Statistical analyses

Statistical analyses were performed using unpaired Student T test with Graph Pad Software.
